# Deep Insight into the Transcriptome of the Single Silk Gland of *Bombyx mori*

**DOI:** 10.3390/ijms20102491

**Published:** 2019-05-20

**Authors:** Run Shi, Sanyuan Ma, Ting He, Jian Peng, Tong Zhang, Xiaoxu Chen, Xiaogang Wang, Jiasong Chang, Qingyou Xia, Ping Zhao

**Affiliations:** 1 Biological Science Research Center, Southwest University, Chongqing 400716, China; shirun186@163.com (R.S.); masy@swu.edu.cn (S.M.); theppone@163.com (J.P.); zt137703197@email.swu.edu.cn (T.Z.); chenxiaoxu95@126.com (X.C.); wangyang217804@126.com (X.W.); jiasongchang@163.com (J.C.); xiaqy@swu.edu.cn (Q.X.); 2 State Key Laboratory of Silkworm Genome Biology, Southwest University, Chongqing 400716, China; hetingar@163.com; 3 Chongqing Engineering and Technology Research Center for Novel Silk Materials, Southwest University, Chongqing 400716, China; 4 Chongqing Key Laboratory of Sericulture, Southwest University, Chongqing 400716, China

**Keywords:** single silk gland, transcriptome, gradually upregulated genes, *Bombyx mori*

## Abstract

The silk gland synthesizes and secretes a large amount of protein and stores liquid silk protein at an extremely high concentration. Interestingly, silk proteins and serine protease inhibitors are orderly arranged in the silk gland lumen and cocoon shells. Silk fiber formation and the spinning mechanism have not been fully elucidated. Therefore, we conducted a comparative transcriptome analysis of seven segments of the single silk gland to characterize internal changes in the silk gland during the 5th instar of mature larvae. In total, 3121 differentially expressed genes were identified in the seven segments. Genes highly expressed in the middle silk gland (MSG) were mainly involved in unsaturated fatty acid biosynthesis, fatty acid metabolism, apoptosis—fly, and lysosome pathways, whereas genes highly expressed in the posterior silk gland (PSG) were mainly involved in ribosome, proteasome, citrate cycle, and glycolysis/gluconeogenesis pathways. Thus, the MSG and PSG differ greatly in energy source use and function. Further, 773 gradually upregulated genes (from PSG to MSG) were involved in energy metabolism, silk protein synthesis, and secretion, suggesting that these genes play an important role in silk fiber formation. Our findings provide insights into the mechanism of silk protein synthesis and transport and silk fiber formation.

## 1. Introduction

Silk is one of the most mysterious and attractive materials in nature and has been widely used in biomedicine, soft-tissue engineering, biosensing, textiles, cosmetics, and other fields. More than 23 categories of insects [1] and at least 47,878 spider species (http://www.wsc.nmbe.ch/) secrete silk. Usually, they secrete silk for cocooning or nesting to protect themselves, or for netting to capture prey. *Bombyx mori* and *Nephila clavipes* are the most studied silk-secreting species. Natural spider silk has extraordinary properties, such as high tensile strength and extensibility. Spiders display aggressive territorial behavior, which renders their large-scale breeding infeasible. Therefore, researchers have made great efforts in developing recombinant spider silk proteins, including the expression of recombinant spider silk proteins in bioreactors such as bacteria, yeast, plants, and transgenic animals. However, these approaches are limited in terms of protein yield, solubility, and stability. The silkworm *B. mori* has been domesticated for over 5000 years. It has significant economic importance and is a model organism for studying lepidopteran and arthropod biology [2,3]. One silkworm with a dry weight of about 2 g can produce up to 500 mg of silk protein in its silk gland, which accounts for approximately 25% of the total silkworm dry weight [4]. Silk proteins can be stored in a soluble form in the silk gland at a very high concentration (up to 25%), without aggregation or denaturalization [5]. This unique protein synthesis and storage capacity provides broad prospects for research on and utilization of the silkworm.

Silk fiber is an ordered composition of silk-associated proteins, including fibroins, sericins, antimicrobial proteins and some proteins of unknown function. Fibroins and sericins are the major components of silkworm silk [6]. Fibroin, which accounts for 70% of silk proteins, is the central fiber protein and is secreted by the posterior silk gland (PSG). Fibroins consists of a fibroin heavy chain (Fib-H, ~350 kDa), fibroin light chain (Fib-L, ~26 kDa), and P25/fibrohexamerin (fhx/P25) at a 6:6:1 molar ratio [7]. The fibroin heavy and light chains are linked by a single disulfide bond, and then combined with P25 by a noncovalent bond [8]. Sericins are soluble glue proteins that coat and cement the silk fibers, and mainly include sericin 1 (Ser1, ~400 kDa), sericin 2 (Ser2, ~230 kDa and 120 kDa), and sericin 3 (Ser3, ~250 kDa) [9,10]. They are secreted by different segments of the middle silk gland (MSG). 

Silk fiber formation is a fascinating process. It is a tightly controlled and dynamic process that takes place within the lumen of the silk gland. Fibroins are secreted by the PSG to form the core structure of the silk fibers. When the silk fibroins are transported to the MSG, Ser1, Ser3, and a small amount of Ser2 are alternately wrapped around the fibroins to form a concentrated aqueous silk solution [11]. During the spinning stage, the mixture moves forward to the anterior silk gland (ASG) and spins out through the ASG and spinneret, accompanied by structural conformational changes. This protein secretion process leads to the orderly composition of silk fibroins and sericins in silk fibers. pH, ions, and shear force are important factors in the silk fiber formation process [12,13]. 

Ser1 is highly expressed in the middle and posterior compartments of the MSG [14], and Ser3 is highly expressed in the anterior segment of the MSG [15]. Protein structural analysis has revealed that Ser3 has stronger hydrophilicity and fluidity than Ser1. This indicates that Ser3 forms the outer silk protein layer, which requires higher fluidity and lower crystallinity to withstand the high shear force in the ASG and spinneret. Ser2 is expressed at a very low level during spinning, and it mainly acts as an adhesive in the silk scaffold [16]. Dong et al. (2016) used liquid chromatography-tandem mass spectrometry (LC-MS/MS) to gain a clear understanding of the proteins in each segment of the silk gland [17]. From day five of the fifth instar to the spinning stage, fibroin H, L, and P25 were increased in the ASG; Ser1, and Ser3 were increased in the ASG and the anterior segment of the MSG, and the serine protease inhibitors BmSPI39 and BmSPI51, and carboxypeptidase inhibitor were mainly increased in the anterior segment of the MSG. The *B. mori* cocoon has a multilayered structure, and by comparing the protein abundance in each layer, Zhang et al. (2015) found that the outermost layer was relatively enriched in Ser1 and protease inhibitors, whereas Ser2 showed very low abundance or was absent in all cocoon layers [18]. 

Although there have been some studies on the orderly composition of silk fibers, the underlying mechanism remains poorly understood. In this study, to better understand the molecular functions of the silk gland subsections and the genes associated with silk protein synthesis and transport, we conducted a comprehensive transcriptome analysis of the single silk gland. Genes highly expressed in the MSG and PSG were identified and compared. Gradually upregulated genes in the silk gland were identified for the first time, and they may possibly be involved in silk protein transport and silk fiber formation. In addition, the peripheral pH in the silk gland was found to change from alkaline to acidic, suggesting intense ion exchange between the lumen of the silk gland and the blood.

## 2. Results

### 2.1. Overall Structure and Analysis of the Transcriptome Data

**Figure 1:**
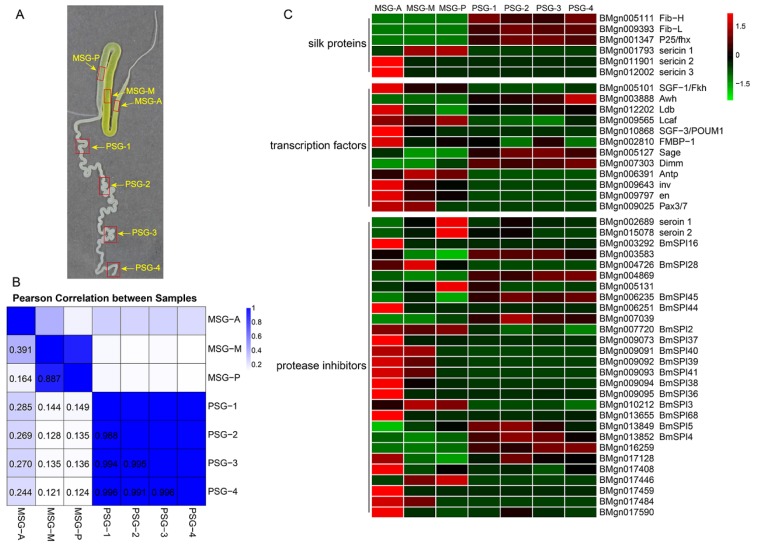
Overview of samples and transcriptome data. (**A**) Silk gland at full maturation of 5th instar. The single silk gland was divided into MSG-A (anterior segment of the middle silk gland), MSG-M (middle segment of the middle silk gland), MSG-P (posterior segment of the middle silk gland), PSG-1, PSG-2, PSG-3, and PSG-4. Red boxes indicate where the samples were collected. (**B**) Pearson correlation analysis of samples. The number is the correlation coefficient between each two samples. (**C**) Heatmap of silk protein-related genes, including silk protein genes, transcription factors, and protease inhibitors.

In this study, the MSG and PSG of the silk gland were divided into seven subsections. The MSG was separated into three subsections; the part proximate to the ASG was labeled MSG-A, the part near the PSG labeled MSG-P, and the middle part was labeled MSG-M. The PSG was divided into four segments, labeled PSG-1, PSG-2, PSG-3, and PSG-4, as shown in Figure 1A. These samples were used for RNA-seq. For each sample, we obtained more than 50 million clean reads, which were nearly 95% of the raw reads (Table S1). Q30 values were >90% in all quality scores and GC contents ranged from 47.28% to 52.18%, suggesting that RNA-Seq data generated in this study were reliable. Meanwhile, 58.56–80.11% and 57.02–75.67% of total reads were mapped to the reference genome and gene using BWA and Bowtie, respectively. Using the FPKM method, we identified 10,190, 9716, and 10,006 expressed genes in the MSG subsections, and 9422, 9438, 9378, 9322 expressed genes in the PSG segments (Table S1). Pearson correlation analysis revealed that all genes identified were classified into three categories, in which the MSG-A formed an independent cluster, whereas MSG-M and MSG-P were clustered together, and the four PSG samples were highly correlated (Figure 1B). This was consistent with findings in previous reports [19,20].

**Figure 2:**
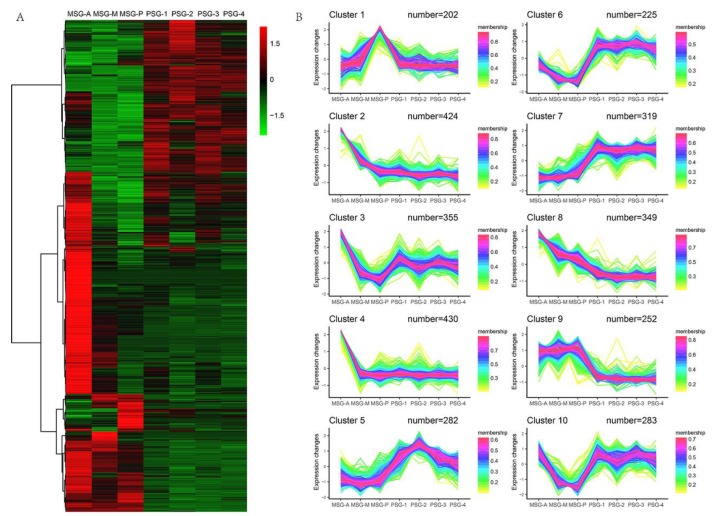
Cluster analysis of all differentially expressed genes (DEGs) between single silk gland samples. (**A**) Hierarchical clustering of DEGs. Red, high expression level; Green, low expression level. DEGs, differentially expressed genes. (**B**) Coexpression analysis of DEGs. Red, high correlation; yellow, low correlation.

To better understand how this obvious classification was produced, differentially expressed gene (DEG) analysis was performed. Genes with FPKM ≥ 5 were considered expressed. FDR ≤ 0.001 and |Log_2_Ratio| ≥ 1 were set as criteria for screening DEGs. Using this approach, 3121 DEGs were identified in the seven sections of the MSG and PSG. We used hierarchical clustering to comparatively visualize the DEGs in the seven samples (Figure 2A). The heatmap indicated that the DEGs mainly exhibited tissue-specific expression in the silk gland, which was in line with the Pearson correlation data. Intriguingly, DEGs in the MSG-A had the highest expression levels compared with those in the other sections. Further coexpression analysis grouped all these DEGs into ten clusters (Figure 2B). DEGs from cluster 1 included 202 MSG-P high-expression genes, DEGs from cluster 4 included 430 MSG-A high-expression genes, DEGs from cluster 5 included 282 PSG-2 high-expression genes, DEGs from clusters 6 and 7 included 544 PSG high-expression genes, DEGs from cluster 9 included 252 MSG high-expression genes, and DEGs from clusters 2 and 8 included 773 gradually upregulated genes. All DEGs were well classified.

### 2.2. Divergent Functions in Each Segment are Related to Silk Protein Synthesis

We first analyzed silk-associated genes in silk glands previously reported, including fibroins, sericins, transcription factors, and protease inhibitors (Figure 1C). *Fib-H*, *Fib-L*, and *P25* were highly expressed at similar levels in the four segments of the PSG, whereas the mRNA expression levels varied greatly among these three genes. The FPKM value of *Fib-L* was one order of magnitude higher than that of *Fib-H* and *P25* (Table S2). According to the results of label-free protein quantification in a previous study, Fib-L and Fib-H have a similar abundance in the cocoon, but are more abundant than P25 [21]. This suggests that *Fib-L* and *P25* produce more redundant RNA than *Fib-H*. *Ser1* was mainly expressed in the middle and posterior areas of the MSG, and at a substantially higher level than *Ser3*. The *Ser2* expression level in the 5th instar of mature larvae was extremely low, and *Ser2* was mainly expressed in the anterior region of the MSG. This is consistent with previous label-free protein quantitative results that revealed that cocoon silk is much more abundant in Ser1 than in the other two sericins. 

Both *SGF-1* and *SGF-3* can regulate *Ser1* expression. Our transcriptome data showed that *SGF-1* was highly expressed in the three segments of MSG, and lowly expressed in the PSG. The high expression region of *SGF-1* contained that of *Ser1*. However, we detected strong *SGF-3/POUM1* expression in the anterior MSG, which was different from the expression region of *Ser1*. Kimoto et al. (2012) reported that *POUM1* combined with the SC site to inhibit *Ser1* expression and thus restricted the anterior boundary of *Ser1* expression [22], our data might supported this finding. Fibroin gene expression is regulated not only by *SGF-2*, but also by *Sage*, *Dimm*, and *FMBP-1*. *SGF-2* is a multisubunit activator complex containing *Awh*, *Ldb*, and *Lcaf*, which control PSG-specific expression of silk fibroin genes [23]. The transcriptome data showed that *Awh* was strictly specifically expressed in the PSG, *Ldb* was highly expressed in the MSG and PSG, and *Lcaf* expression was lower in the silk gland (Table S2). *Bmsage* and *Bmdimm* were both highly expressed in the MSG and PSG, but the expression levels were higher in the PSG. *FMBP-1* was expressed in both the MSG and PSG, at low levels. These six transcription factors were all expressed in the PSG. Furthermore, the four transcription factors *Antp*, *invected (inv)*, *engrailed (en)*, and *paired box (Pax)* were expressed in the MSG, but not in the PSG. Protease inhibitors are very abundant in the silk gland. We detected 28 protease inhibitors that differed between the MSG and PSG, and their expression regions were obviously different. It was found that the two seroin proteins (seroin 1 and seroin 2) were highly expressed in the MSG and PSG. Serine protease inhibitors are the most diverse and abundant protease inhibitors in the silk gland. The expression levels of BmSPI38, BmSPI39, BmSPI40, and BmSPI41 showed a gradient distribution, whereas BmSPI16 had a specific and high expression level in the anterior MSG. This is consistent with the previously reported gradient distribution of serine protease inhibitors in the cocoon shell, which enables silkworm pupae to resist infection [18].

### 2.3. The MSG is Functionally More Divergent than the PSG

**Figure 3:**
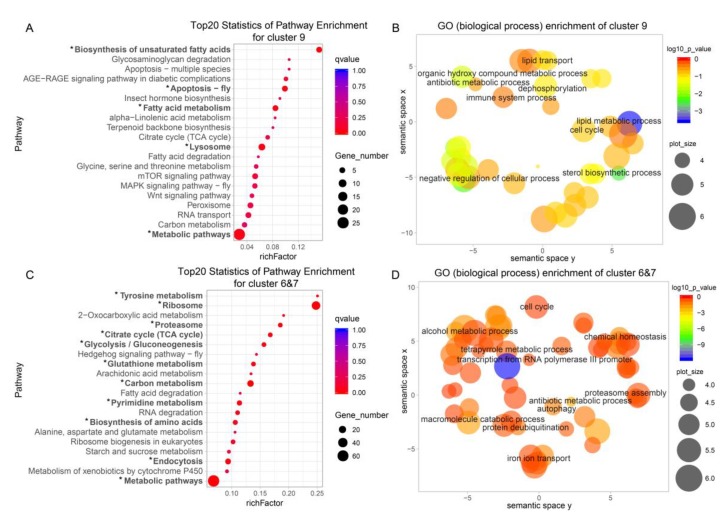
Kyoto Encyclopedia of Genes and Genomes (KEGG) pathway enrichment analysis and gene ontology (GO) classification of high expression genes in the MSG and PSG. (**A**,**C**) KEGG pathway enrichment analysis of high expression genes in the MSG (**A**) and PSG (**C**), respectively. “*” (qvalue < 0.05) represents a significantly enriched pathway. MSG, middle silk gland; PSG, posterior silk gland. (**B**,**D**) GO scatterplot constructed with REVIGO for high expression genes in the MSG (**B**) and PSG (**D**), respectively. Colors represent the *p*-value according to the legend. Bubble size reflects the frequency of each GO term.

Pearson correlation and the heatmap (Figure 1B, Figure 2A) revealed that gene expression in the PSG was quite consistent, whereas that in the MSG was more diverse. To obtain a concise and visual GO dataset with no redundant terms, we made use of REVIGO [24], which allows clustering of semantically similar GO terms and labeling each cluster with a single representative GO term. This makes it easier to discover biologically relevant gene groups. Cluster 9 and clusters 6 & 7 represent MSG and PSG high-expression genes, respectively (Figure 2B). Genes highly expressed in the MSG were mainly involved in lipid metabolism, lipid transport, negative regulation of cell processes, dephosphorylation, and antibiotic metabolism (Figure 3B). It was very interesting to find genes related to lipid metabolism and lipid transport to be highly expressed in MSG. This indicates that the energy for the secretion and transport of silk protein not only comes from sugar metabolism, but also from lipid metabolism. We identified nine lipid metabolism-related genes and one lipid transport-related gene in the MSG high-expression group. Among them, BMgn009790 (carotenoid-binding protein (Cbp)), BMgn012648 (lipase 1), and BMgn008405 (ecdysteroid-regulated 16-kDa protein precursor) had relatively high expression levels. Genes highly expressed in the PSG were mainly involved in macromolecule catabolism, alcohol metabolism, vesicle-mediated transport, chemical homeostasis, and proteasome assembly, and thus, in protein synthesis and degradation (Figure 3D). Two genes (BMgn006175, ATG4B, and BMgn017054, ATG6) involved in autophagy were enriched.

KEGG analysis produced results very similar to those obtained with REVIGO. In cluster 9, the biosynthesis of unsaturated fatty acids was the most enriched, and fatty acid metabolism and fatty acid degradation were also enriched (Figure 3A). In clusters 6 & 7, tyrosine metabolism and ribosome pathways were the most abundant (Figure 3C). In addition, the most enriched pathways in cluster 9 included apoptosis—fly, lysosome, and MAPK signaling pathway, which are related to apoptosis. The combination of REVIGO analysis and KEGG analysis demonstrated that the MSG is more inclined toward lipid metabolism, whereas the PSG is more inclined toward amino acid metabolism in the fifth-instar mature larva. 

### 2.4. Specific Genes Contribute to the Distinct Functions of the Anterior MSG

**Figure 4:**
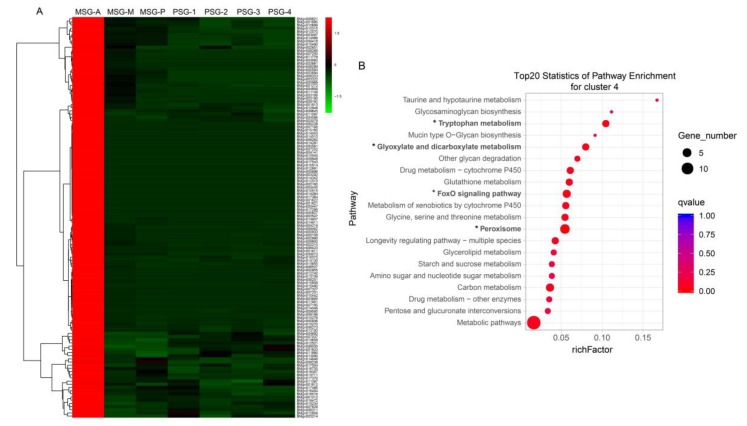
Cluster and KEGG enrichment analysis of MSG-A highly expressed genes. (**A**) Hierarchical clustering of high expression genes in the MSG-A. (**B**) KEGG pathway enrichment analysis of high expression genes in the MSG-A. “*” (qvalue < 0.05) represents a significantly enriched pathway.

The genes in cluster 4 showed distinct characteristics, with specific and high-level expression in the anterior MSG. We screened the cluster 4 genes according to fold change ≥ 2 in MSG-A versus the other samples and gene expression levels of other samples < 5. Thus, 126 genes that were specifically and highly expressed in the MSG-A were detected (Figure 4A, Table S3). KEGG pathway enrichment analysis of these 126 genes revealed that tryptophan metabolism, glyoxylate and dicarboxylate metabolism, FoxO signaling pathway, and peroxisome pathways were significantly enriched (Figure 4B). Three catalases (BMgn012691, BMgn014284, BMgn014510) were involved in these four significantly enriched pathways. Four cytochrome P450 genes (BMgn007168, BMgn007195, BMgn014043, BMgn014046), all of which belong to the CYP4 family, were identified to be specifically and highly expressed in MSG-A. Five serine protease inhibitors, including BMgn003292, BMgn006251, BMgn009073, BMgn009095, BMgn013655, were specifically expressed in the anterior MSG. Among them, BMgn003292 (BmSPI16) contains the serpin domain, BMgn006251 (BmSPI44), BMgn009073 (BmSPI37), BMgn009095 (BmSPI36) contain the TIL domain, and BMgn013655 (BmSPI68) contains the kazal domain, which may be involved in resistance to pathogenic microorganisms [25]. Six genes (BMgn006273, BMgn007037, BMgn009192, BMgn009198, BMgn013708, BMgn015270) involved in ion and small-molecule transport were detected. Ecdysone oxidase (BMgn000158) was also strongly expressed in the anterior MSG. It is a key enzyme that converts ecdysone into 3-dehydroecdysone and inactivates ecdysone [26].

### 2.5. Gradually Upregulated Genes Contribute to Silk Protein Storage and Silk Formation

**Figure 5:**
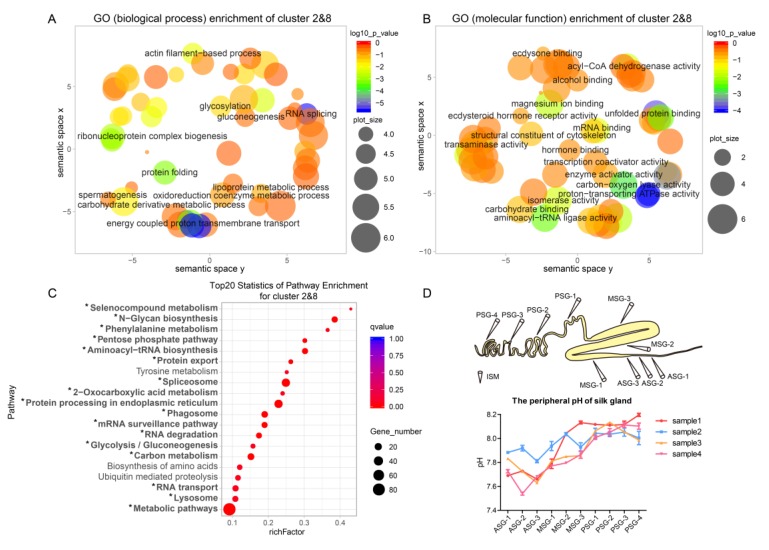
Functional analysis of gradually upregulated genes. (**A**,**B**) GO scatterplot constructed with REVIGO for gradually upregulated genes related to biological process (**A**) and molecular function (**B**). (**C**) KEGG pathway enrichment analysis of gradually upregulated genes in cluster 2 and cluster 8. “*” (qvalue < 0.05) represents a significantly enriched pathway. (**D**) Detection of the peripheral pH of the silk gland by ion-selective microelectrode (ISM).

One group of genes, including clusters 2 and 8, showed an increasing expression trend from the PSG to the MSG; we refer to them as gradually upregulated genes here. For functional annotation of the genes in clusters 2 and 8, we used GO enrichment analysis. In total, 229 GO terms were assigned. In “biological process,” DEGs were mainly involved in RNA splicing, ribonucleoprotein complex biogenesis, protein folding, actin filament-based process, in which energy-coupled proton transmembrane transport was the most significantly enriched (Figure 5A). In “molecular function”, the GO terms were enriched in proton-transporting ATPase activity, transaminase activity, unfolded protein binding, magnesium ion binding, and acyl-CoA dehydrogenase activity, while proton-transporting ATPase activity was the most significantly enriched (Figure 5B). 

To identify the biological pathways involved in the process of silk protein secretion, KEGG enrichment analysis was employed. We identified 98 pathways, 17 of which were significantly enriched (qvalue < 0.05; Figure 5C). These 17 pathways included three energy metabolism-related pathways (2-oxocarboxylic acid metabolism, glycolysis/gluconeogenesis, carbon metabolism), six protein synthesis-related pathways (phenylalanine metabolism, aminoacyl-tRNA biosynthesis, spliceosome, RNA transport, mRNA surveillance pathway, RNA degradation), and two protein secretion-related pathways (N-glycan biosynthesis, protein export). These results suggested that protein synthesis and secretion are major activities in the silk gland during the 5th instar of mature larvae stage.

Proton-transporting ATPases were abundant in the silk gland as indicated by the GO enrichment analysis. Seven vacuolar-type proton ATPase (V-ATPase) genes with a gradually upregulated expression pattern were identified (Table S4). Proton-transporting ATPases maintain the acidic environment of the silk gland, which is crucial for silk fiber formation, by producing H^+^ [27]. We measured the peripheral pH of the silk gland using an ion-selective microelectrode technique. A pH gradient from the peripheral PSG to the ASG was observed (Figure 5D). The peripheral pH of the PSG was between 8.05 and 8.2, which is alkaline. The peripheral pH of the MSG and the beginning of ASG ranged from 7.8 to 7.6, which is slightly alkaline. Since the silk gland needs ion exchange with the blood to cause the pH change of the silk gland, it is speculated that the lumen of the silk gland has intense ion change with the blood, which leads to a dramatic pH change in the silk gland lumen.

**Figure 6:**
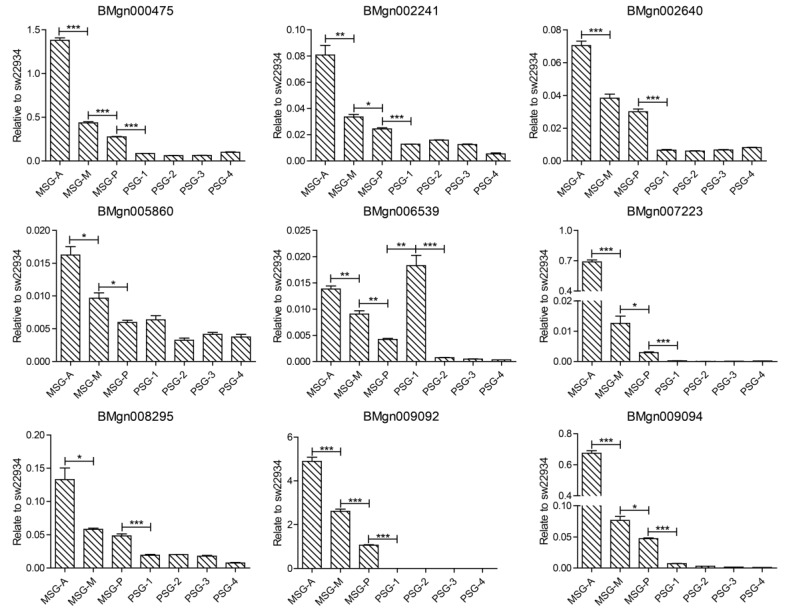
Quantitative PCR verification of partial gradually upregulated genes. “*” stands for *p*-value < 0.05, “**” stands for *p*-value < 0.01, and “***” stands for *p*-value < 0.001.

Nine genes with gradually upregulated expression pattern were verified by quantitative RT-PCR; the expression levels of all nine genes gradually increased from the PSG to the MSG, and the major expression differences were observed in the three subsegments of MSG (Figure 6). This result confirmed that the transcriptome sequencing data were credible. Among the genes tested, BMgn002241 (vacuolar ATPase subunit B) and BMgn008295 (vacuolar ATPase subunit A) are two subunits of V-ATPase. BMgn000475 (calreticulin, CRT) is expressed in the fat body, midgut, silk gland, ovary, and testes of *B. mori*. The highest CRT expression is detected in the fat body [28], and CRT expression is higher in the MSG than the PSG [29]. BMgn005860 (reticulon-1-like, RTNL1) is a member of the RTN family and participates in the secretory pathways of the endoplasmic reticulum and the Golgi apparatus. BMgn006539 (angiotensin-converting enzyme-like, ACE) is highly expressed in the silk gland and testes.

## 3. Discussion

In this study, we used RNA-Seq to evaluate transcriptional differences in various single silk gland segments. Our results revealed that DEGs in the different segments of the silk gland tend to have distinct functions, and we identified gradually upregulated genes possibly involved in silk protein transport and silk fiber formation.

The orderly composition is largely related to their secretory regions, in which transcription factors play an important role. This study revealed expression relationships between transcription factors and silk protein genes (Figure 1C). Silk protein synthesis reached its peak at the 5th instar of mature larvae. At this time, *SGF-1* and *POUM1* together determined the expression region of *Ser1* in the MSG-M and MSG-P [22,30], The expression region of *SGF-1* and *POUM1*, with that of *Ser1*, may also indirectly prove this view. *SGF-1/Fkh*, *Awh*, *FMBP-1*, *Bmsage*, and *Bmdimm* had been shown to be involved in the transcriptional regulation of fibroin genes [31,32,33,34,35], but only *Awh* and *Bmdimm* presented a consistent spatial and temporal expression pattern with the fibroin genes [36]. *Awh* was specifically expressed in the PSG, and misexpression of *Awh* induced the ectopic expression of the fibroin genes in the MSG [32]. Compared with other transcription factors, the expression level of *Awh* was extremely low. It suggested that *Awh* with low expression level still had a key regulatory effect on the expression of fibroin genes. *Antp*, *inv*, *en*, and *Pax* were only expressed in the MSG. It had been reported that *Antp* regulated *Ser1* expression [37], and *en* and *inv* may be involved in silk gland differentiation [38]. The function of these transcription factors in the silk gland remains to be further studied. Our data suggested that tissue-specific *sericin* and *fibroin* expression is a result of interactions of multiple transcription factors. 

Protease inhibitors are abundant and important in the silk gland. We identified 28 protease inhibitors in total. Some of them were highly expressed in the MSG and PSG, some were gradually upregulated, and some were specifically and highly expressed in the MSG-A. Seroin 1 and seroin 2 were glycoproteins with a molecular weight of 8 kDa and 13 kDa, respectively [39]. Seroin 2 inhibited the growth of Gram-positive and Gram-negative bacteria, whereas seroin 1 only inhibited the growth of Gram-positive bacteria [40]. BMgn009092 (BmSPI39) and BMgn009094 (BmSPI38) were two serine protease inhibitors with a TIL domain that strongly inhibited microbial-derived subtilisin and proteinase K, and potently inhibited the virulence protease CDEP-1, protecting against *Beauveria bassiana* invasion [41,42]. BmSPI16 and BmSPI44 had very high expression levels, and they were specifically and highly expressed in the MSG-A. BmSPI16 (serpin16) strongly inhibits cysteine proteases, such as papain and cathepsin L [43]. BmSPI16 and BmSPI44 are abundant in the outermost layer of the cocoon shell [18], and protect the cocoon shell against bacterial and fungal invasion. The segmental expression pattern of protease inhibitors in the silk gland was also related to their distribution in the cocoon shell. The outer layer contains more protease inhibitors than the inner layer, which means it can provide better protection for pupae [18]. The abundant protease inhibitors in the silk gland bring a wide range of antibacterial activity and provide sufficient protection for the pupa.

The MSG and PSG differ greatly in morphology and function, whereas gene expression differences were more obvious between the MSG-A and other segments. In terms of energy metabolism, the common pathways of the MSG and PSG are carbon metabolism and citrate cycle (TCA cycle). Further, MSG also utilized fatty acid metabolism, and the PSG utilized glycolysis/gluconeogenesis. The biosynthesis of unsaturated fatty acids and fatty acid metabolism in the MSG were new discoveries in this study. Nine lipid metabolism-related genes and one lipid transport-related gene were identified to be highly expressed in the MSG. Carotenoid binding protein, which contains a lipid-binding domain known as the steroidogenic acute regulatory protein-related lipid transfer domain, determines the color (yellow) of the blood, silk gland, and cocoon of *B. mori* [44,45]. BMgn012648 (lipase 1) was the most strongly expressed, and it was not expressed in the PSG. FoxO overexpression upregulates the expression of acid lipase-1 and promotes lipolysis in fat body [46]. We speculate that lipase-1 also undergoes lipolysis through a similar pathway in the MSG, providing energy for spinning. BMgn008405 (ecdysteroid-regulated 16-kDa protein precursor) was strongly expressed in both the MSG and the PSG, but higher in the MSG. Ecdysteroids are precursors of 20-hydroxyecdysone (20E), which play a coordinating role in insect molting and metamorphosis. 20E can induce tissue morphological changes in the absence of juvenile hormone (JH). This suggests that dramatic changes take place in the silk gland during larval maturation. Numerous genes involved in ribosome, biosynthesis of amino acids, and tyrosine metabolism pathways were identified in the PSG, which means protein synthesis is still occurring at large scale in the PSG during larval maturation. This may be related to the large proportion of silk fibroin in the cocoon shell. 

The MSG-A had more DEGs, suggesting it had unique functions. The acidification of the silk gland lumen starts mainly from the anterior MSG, and the inner diameter of the silk gland lumen also gradually decreases from the anterior MSG. *Ser2* is specifically expressed in the MSG-A, and accordingly, Ser2 is only present in the silk scaffold and plays an important role in the adhesion of cocoon shell. Catalase plays an important role in facilitating the degradation of H_2_O_2_ to alleviate oxidative stress. It is widely distributed in all tissues of *B. mori* larvae, although the expression level is relatively low in the testes and ovary. In a study by Yamamoto et al. (2005) the optimum pH for recombinant *B. mori* catalase was 8.0 and the optimum temperature was 20–40 °C. At pH 7, the catalase still had more than 80% activity [47]. The pH of the anterior MSG of *B. mori* is 6.8–7.0, thus, catalase activity is quite high. P450s act in the detoxification of plant allelochemicals and insecticides, while the CYP3 family of *B. mori* reportedly is involved in the ecdysteroid synthesis pathways [48,49]. EO degrades ecdysteroids, and a low level of ecdysteroids contributes to silk protein synthesis. Sun et al. considered that BMgn000158 does not have EO activity because it has only one conserved ecdysone-binding residue. Therefore, the function of EO specifically expressed in the MSG-A remains to be explored [26].

Natural silk spinning is nucleation-dependent, which depends on the silk protein solution, pH gradient, metallic ion content, shear force [3]. H^+^ is crucial for generating a pH gradient [50]. In *N. clavipes*, the pH gradient is created by carbonic anhydrase in the major ampullate gland, which catalyzes the conversion of H_2_O and CO_2_ in the gland lumen to H^+^ and HCO_3_^-^, causing a change of pH in the major ampullate gland lumen [51]. In *B. mori*, carbonic anhydrase also regulates the pH gradient change of the silk gland from alkaline to acidic, and after the addition of the carbonic anhydrase inhibitor (methazolamide), the pH of all positions in the silk gland lumen becomes neutral [52]. However, we detected low carbonic anhydrase expression in the MSG and PSG, without gradual upregulation in the different segments. In addition to carbonic anhydrase, v-ATPase also regulates the pH of the silk gland. However, by simply inhibiting the activity of carbonic anhydrase in the silk gland, the pH of the silk gland lumen becomes neutral. This suggests that carbonic anhydrase plays an important role in maintaining the pH of the silk gland, perhaps as an intermediate bridge between v-ATPase and other pH-regulating factors. As can be seen from the data of Chang et al. (2015), carbonic anhydrase has a relatively high expression level in the ASG. The pH gradient in the ASG has a crucial influence on the conformational transformation of silk fibroin, and it is foreseen that it will have a significant effect on silk formation in the absence of carbonic anhydrase in the individuals. This will provide a basis for research on the silk secretion mechanism of *B. mori*. 

H^+^ transport-related genes are also involved in silk gland pH regulation. V-type ATPase is usually expressed on the plasma membrane or endometrium system and uses the energy generated by ATP decomposition to pump the H^+^ against the concentration gradient into the cell lumen to maintain the intracellular pH, or to pump H^+^ out to acidify the external microenvironment [53]. In a study by Azuma and Ohta (1998), when silk glands were treated with bafilomycin A_1_, a specific inhibitor of V-ATPase, acidification was completely but reversibly inhibited, indicating that V-ATPase participates in silk gland pH regulation [27]. Chang et al. (2015) reported that in the ASG, 18 genes were enriched in ion-transporting pathways, 15 of which encoded different subunits of V-ATPase [19]. Comparative transcriptome analysis of spinnerets and Filippi’s glands revealed elevated expression of large numbers of V-ATPase genes and proton transporter genes in the spinneret, 12 among which were V-ATPase subunits [54]. We found 17 highly expressed V-ATPase genes in the silk gland, seven of which were gradually upregulated (Table S4). By comparison with existing transcriptome data, it was found that the total number of V-ATPases identified was similar, and more than half of the genes identified were the same as in previous studies. V-ATPase is also involved in silkworm immunity, and transient overexpression of the V-ATPase C subunit in BmNPV-infected silkworm cells can significantly inhibit BmNPV proliferation [55]. This may be due to blockage of viral entry into the cells, or by speeding up proton transport, resulting in rapid acidification of the target membrane organelles and effective degradation of the invading virus [55]. We found that the peripheral pH of the silk gland decreased from 8.2 (the highest) to 7.6 (the lowest) from the PSG to the MSG. This suggested that the silk gland needs to exchange hydrogen ions with the outside environment to regulate the intraluminal pH. 

A large number of gradually upregulated genes were involved in energy metabolism, silk protein synthesis and secretion, and other pathways involved in silk fiber formation. The three energy metabolism-related pathways contained 20 genes, the six silk protein synthesis-related pathways contained 74 genes, and the two silk protein transport-related pathways contained 19 genes. The large number of gradually upregulated genes suggests that they play important roles in silk protein synthesis and transport as well as in silk fiber formation. Calreticulin (CRT) plays an important role in chaperoning and regulating Ca^2+^ homeostasis in the ER lumen, which is an important organelle for processing proteins. CRT is a lectin-like chaperone, which is involved in the quality control of the synthesis process of ion channels, surface receptors, integrins, and transporters. It can prevent the aggregation of partially folded proteins and improve the yield and assembly rate of correctly folded proteins [56]. Reticulons are highly conserved ER membrane proteins that promote high curvature smooth ER tubules in the cell cortex [57]. Functional deletion mutation of RNTL1 in *Drosophila melanogaster* resulted in a significant decrease in life expectancy [58]. In *B. mori*, there have been few reports about the molecular function of CRT and RTNL1 in recent years. The most important function of the silk gland is to synthesize silk protein. Studying the function of CRT and RTNL1 in the silk gland might play a crucial role in increasing silk protein production. Angiotensin-converting enzyme (ACE) is highly expressed in the testis and silk gland of *B. mori*. The study of ACE in insects is mostly focused on its effects on metamorphosis and reproduction [59]. However, high levels of ACE expressed in the silk gland may not be associated with metamorphosis and reproduction. In mammals, ACE is involved in the regulation of blood pressure through the renin–angiotensin system [60]. The silk gland is also a lumen structure, and ACE might play a role in regulating the pressure within the silk gland lumen. The rapid development of genome editing technology has greatly facilitated the study of gene function. Genomic editing tools will play an important role in studying the function of these candidate genes in *B. mori*.

## 4. Materials and Methods

### 4.1. Silkworm

*B. mori* strain Dazao were supplied by the State Key Laboratory of Silkworm Genome Biology, Southwest University (Chongqing, China). The silkworms were reared at 25 °C with fresh mulberry leaves.

### 4.2. Silk Gland Dissection and Sample Collection

Silkworms of 5th larvae at full maturation were used. Silk glands were dissected in phosphate-buffered saline (pH 7.4), and the middle part of the three subsections of the MSG and the adjacent regions of the PSG at the same distance were collected. To ensure uniformity, samples were collected from the same single silk gland.

### 4.3. RNA Preparation and Transcriptome Sequencing

The collected samples were homogenized in a Micro Smash disrupter (Tomy, Japan). Total RNA was extracted using a Total RNA Kit II (Omega Bio-tek, Nrocross, GA, USA). RNA quality and quantity were measured on an Agilent Bioanalyzer 2100 system (Agilent Technologies, Santa Clara, CA, USA). Transcript library construction and sequencing were performed at BGI-Tech, Beijing. In brief, 3 μg RNA per sample was used for mRNA-seq library construction. After total RNA was digested with DNase I, mRNA was enriched using oligo(dT)-decorated magnetic beads. The mRNA was fragmented into short fragments, which were then transcribed into first- and second-strand cDNAs. Following purification, end reparation, single nucleotide A (adenine) addition, adapter ligation, and suitable fragment selection, the libraries were generated by PCR. The Agilent 2100 Bioanalyzer was used for quality control during sample library assessment. Finally, the libraries were sequenced on Illumina HiSeq^TM^ 2000 instrument.

### 4.4. Sequencing Data Processing

Raw reads in fastq format were processed to obtain clean reads by removing reads containing adapter, reads containing more than 10% of poly-N, and low-quality reads. The Q20, Q30, and the GC content of the clean data were calculated. All downstream analyses were based on the high-quality clean data. The *B. mori* reference genome and gene model annotation files were directly downloaded from the silkworm genome database (KAIKObase: http://sgp.dna.affrc.go.jp/pubdata/genomicsequences.html). Clean reads were aligned to the reference genome using the Burrows–Wheeler Alignment tool (BWA) and Bowtie; only perfect and unique matches were retained. Gene and isoform expression levels were quantified using RSEM (RNA-seq by expectation maximization) and the FPKM (fragments per kilobase of transcript per million mapped fragments). For each sequenced library, FPKM normalization steps were included to ensure that expression levels of different genes and transcripts could be compared across runs. Clean data presented in this paper have been deposited in the NCBI Short Read Archive (http://www.ncbi.nlm.nih.gov/sra/) with the accession number PRJNA533027.

### 4.5. Differential Expression Analysis

Differentially expressed genes (DEGs) were screened based on Poisson distribution analysis. *P*-values were adjusted for multiple testing using the Benjamini–Hochberg method. A false discovery rate (FDR) ≤ 0.001 and |Log_2_Ratio| ≥ 1 were used as thresholds to judge the significance of a gene expression difference. The DEGs were subjected to GO enrichment analysis; GO terms with a corrected *P*-value < 0.05 were considered significantly enriched. GO enrichment was obtained by clusterProfiler [61] and visualization with REVIGO [24]. Significantly enriched KEGG pathways were identified with KOBAS 3.0 software, and pathways with qvalue < 0.05 were defined as enriched. Hierarchical clustering of gene expression was performed by pheatmap [62]. Gene coexpression analysis was achieved by Tcseq [63].

### 4.6. Quantitative Reverse Transcription PCR (qRT-PCR)

Total RNA from the subsections of silk gland of 5th larvae at full maturation were extracted using TRIzol^TM^ (Invitrogen, USA) and 1 μg total RNA was reverse-transcribed using the PrimeScript^TM^ RT Reagent Kit with gDNA Eraser (Takara, Japan). The silkworm transcription initiation factor 4a gene (*Tif4a*) was used as an internal control. Primer sets for genes targeted for gene expression validation were designed using Primer-premier 5 software (PREMIER Biosoft) and were synthesized by BGI. The primers are listed in Table S5. Gene relative expression levels were determined by the 2^–∆∆Ct^ method. Three independent replicates were included. 

### 4.7. pH Measurement of the Silk Gland Peripheral

Ion-selective microelectrodes were used to measure the concentration of H^+^ of the silk gland peripheral. The ion selective microelectrode (ISM) consists of three parts, a glass microelectrode, a liquid ion exchanger (LIX), and a backfilling solution. It was first packaged with a backfilling solution (15 mM NaCl, 40 mM KH_2_PO_4_, pH 7.0) to the length of about 1.0 cm from the tip. A 40–50 um H^+^ selective LIX (XY-SJ-H, Xuyue, Beijing, China) was filled in the front of the glass microelectrode. The Ag/AgCl wire microsensor holder YG003-Y11 (Younger USA) was inserted into the back of the ISM to make electrical contact with the electrolyte solution. YG003-Y11 (Younger, Amherst, MA, USA) was used as a reference microsensor. The H^+^ selective microelectrode was calibrated with standard solutions of pH 9.0, 7.0, and 5.0.

After the silk gland was dissected, it was first incubated in a spider ringer to equilibrate for 5 min before starting the test. A total of four silk glands were measured, and sample 1, 2, 3, 4 indicated the four silk glands respectively. Four points were selected in the PSG and one point was selected in the anterior, middle, and posterior areas of the MSG, respectively. Since the ASG was too thin, three points were selected at the front of the ASG as far as possible. Each point was measured at least 20 times. The pH we measured was the peripheral pH of the silk gland, not the pH inside the silk gland lumen. The potential difference between the peripheral of the silk gland and the spider ringer was recorded, and converted into a change in H^+^ concentration according to the Nernst equation. The original data of pH measured by ISM can be found in Table S6.

## Supplementary Materials

Supplementary materials can be found at https://www.mdpi.com/1422-0067/20/10/2491/s1.

## Abbreviations

ASG	Anterior silk gland
DEG	Differentially expressed gene
FDR	False discovery rate
MSG	Middle silk gland
PSG	Posterior silk gland

## References

[1] Sutherland T.D., Young J.H., Weisman S., Hayashi C.Y., Merritt D.J. (2010). Insect silk: One name, many materials. Annu. Rev. Entomol..

[2] Goldsmith M.R., Shimada T., Abe H. (2005). The genetics and genomics of the silkworm, *Bombyx mori*. Annu. Rev. Entomol..

[3] Xia Q., Li S., Feng Q. (2014). Advances in silkworm studies accelerated by the genome sequencing of *Bombyx mori*. Annu. Rev. Entomol..

[4] Ma S., Shi R., Wang X., Liu Y., Chang J., Gao J., Lu W., Zhang J., Zhao P., Xia Q. (2014). Genome editing of *BmFib-H* gene provides an empty *Bombyx mori* silk gland for a highly efficient bioreactor. Sci. Rep..

[5] Lin Z., Huang W., Zhang J., Fan J.S., Yang D. (2009). Solution structure of eggcase silk protein and its implications for silk fiber formation. Proc. Natl. Acad. Sci. USA.

[6] Gamo T., Inokuchi T., Laufer H. (1977). Polypeptides of fibroin and sericin secreted from the different sections of the silk gland in *Bombyx mori*. Insect Biochem..

[7] Inoue S., Tanaka K., Arisaka F., Kimura S., Ohtomo K., Mizuno S. (2000). Silk fibroin of *Bombyx mori* is secreted, assembling a high molecular mass elementary unit consisting of H-chain, L-chain, and P25, with a 6:6:1 molar ratio. J. Biol. Chem..

[8] Tanaka K., Mori K., Mizuno S. (1993). Immunological identification of the major disulfide-linked light component of silk fibroin. J. Biochem..

[9] Takasu Y., Hata T., Uchino K., Zhang Q. (2010). Identification of ser2 proteins as major sericin components in the non-cocoon silk of *Bombyx mori*. Insect Biochem. Mol. Biol..

[10] Takasu Y., Yamada H., Tsubouchi K. (2002). Isolation of three main sericin components from the cocoon of the silkworm, *Bombyx mori*. Biosci. Biotechnol. Biochem..

[11] Shao Z., Vollrath F. (2002). Surprising strength of silkworm silk. Nature.

[12] He Y.X., Zhang N.N., Li W.F., Jia N., Chen B.Y., Zhou K., Zhang J., Chen Y., Zhou C.Z. (2012). N-terminal domain of *Bombyx mori* fibroin mediates the assembly of silk in response to pH decrease. J. Mol. Biol..

[13] Greving I., Cai M., Vollrath F., Schniepp H.C. (2012). Shear-induced self-assembly of native silk proteins into fibrils studied by atomic force microscopy. Biomacromolecules.

[14] Liu Y., Yu L., Guo X., Guo T., Wang S., Lu C. (2006). Analysis of tissue-specific region in sericin 1 gene promoter of *Bombyx mori*. Biochem. Biophys. Res. Commun..

[15] Takasu Y., Yamada H., Tamura T., Sezutsu H., Mita K., Tsubouchi K. (2007). Identification and characterization of a novel sericin gene expressed in the anterior middle silk gland of the silkworm *Bombyx mori*. Insect Biochem. Mol. Biol..

[16] Kludkiewicz B., Takasu Y., Fedic R., Tamura T., Sehnal F., Zurovec M. (2009). Structure and expression of the silk adhesive protein ser2 in *Bombyx mori*. Insect Biochem. Mol. Biol..

[17] Dong Z., Zhao P., Zhang Y., Song Q., Zhang X., Guo P., Wang D., Xia Q. (2016). Analysis of proteome dynamics inside the silk gland lumen of *Bombyx mori*. Sci. Rep..

[18] Zhang Y., Zhao P., Dong Z., Wang D., Guo P., Guo X., Song Q., Zhang W., Xia Q. (2015). Comparative proteome analysis of multi-layer cocoon of the silkworm, *Bombyx mori*. PLoS ONE.

[19] Chang H., Cheng T., Wu Y., Hu W., Long R., Liu C., Zhao P., Xia Q. (2015). Transcriptomic analysis of the anterior silk gland in the domestic silkworm (*Bombyx mori*) - insight into the mechanism of silk formation and spinning. PLoS ONE.

[20] Li J.Y., Ye L.P., Che J.Q., Song J., You Z.Y., Yun K.C., Wang S.H., Zhong B.X. (2015). Comparative proteomic analysis of the silkworm middle silk gland reveals the importance of ribosome biogenesis in silk protein production. J. Proteom..

[21] Dong Z., Zhao P., Wang C., Zhang Y., Chen J., Wang X., Lin Y., Xia Q. (2013). Comparative proteomics reveal diverse functions and dynamic changes of *Bombyx mori* silk proteins spun from different development stages. J. Proteome Res..

[22] Kimoto M., Kitagawa T., Kobayashi I., Nakata T., Kuroiwa A., Takiya S. (2012). Inhibition of the binding of msg-intermolt-specific complex, MIC, to the sericin-1 gene promoter and sericin-1 gene expression by *POU-M1*/*SGF-3*. Dev. Genes Evol..

[23] Ohno K., Sawada J., Takiya S., Kimoto M., Matsumoto A., Tsubota T., Uchino K., Hui C.C., Sezutsu H., Handa H. (2013). Silk gland factor-2, involved in fibroin gene transcription, consists of LIM homeodomain, LIM-interacting, and single-stranded DNA-binding proteins. J. Biol. Chem..

[24] Supek F., Bosnjak M., Skunca N., Smuc T. (2011). REVIGO summarizes and visualizes long lists of gene ontology terms. PLoS ONE.

[25] Zhao P., Dong Z., Duan J., Wang G., Wang L., Li Y., Xiang Z., Xia Q. (2012). Genome-wide identification and immune response analysis of serine protease inhibitor genes in the silkworm, *Bombyx mori*. PLoS ONE.

[26] Sun W., Shen Y.H., Qi D.W., Xiang Z.H., Zhang Z. (2012). Molecular cloning and characterization of ecdysone oxidase and 3-dehydroecdysone-3alpha-reductase involved in the ecdysone inactivation pathway of silkworm, *Bombyx mori*. Int. J. Biol. Sci..

[27] Azuma M., Ohta Y. (1998). Changes in H^+^-translocating vacuolar-type ATPase in the anterior silk gland cell of *Bombyx mori* during metamorphosis. J. Exp. Biol..

[28] Goo T.W., Park S., Jin B.R., Yun E.Y., Kim I., Nho S.K., Kang S.W., Kwon O.Y. (2005). Endoplasmic reticulum stress response of *Bombyx mori* calreticulin. Mol. Biol. Rep..

[29] Hou Y., Xia Q., Zhao P., Zou Y., Liu H., Guan J., Gong J., Xiang Z. (2007). Studies on middle and posterior silk glands of silkworm (*Bombyx mori*) using two-dimensional electrophoresis and mass spectrometry. Insect Biochem. Mol. Biol..

[30] Mach V., Takiya S., Ohno K., Handa H., Imai T., Suzuki Y. (1995). Silk gland factor-1 involved in the regulation of *Bombyx* sericin-1 gene contains fork head motif. J. Biol. Chem..

[31] Julien E., Bordeaux M.C., Garel A., Couble P. (2002). Fork head alternative binding drives stage-specific gene expression in the silk gland of *Bombyx mori*. Insect Biochem. Mol. Biol..

[32] Kimoto M., Tsubota T., Uchino K., Sezutsu H., Takiya S. (2015). LIM-homeodomain transcription factor *Awh* is a key component activating all three fibroin genes, *fibH*, *fibL* and fhx, in the silk gland of the silkworm, *Bombyx mori*. Insect Biochem. Mol. Biol..

[33] Takiya S., Kokubo H., Suzuki Y. (1997). Transcriptional regulatory elements in the upstream and intron of the fibroin gene bind three specific factors *POU-M1*, *Bm Fkh* and *FMBP-1*. Biochem J..

[34] Zhao X.M., Liu C., Li Q.Y., Hu W.B., Zhou M.T., Nie H.Y., Zhang Y.X., Peng Z.C., Zhao P., Xia Q.Y. (2014). Basic helix-loop-helix transcription factor *Bmsage* is involved in regulation of fibroin h-chain gene via interaction with sgf1 in *Bombyx mori*. PLoS ONE.

[35] Zhao X.M., Liu C., Jiang L.J., Li Q.Y., Zhou M.T., Cheng T.C., Mita K., Xia Q.Y. (2015). A juvenile hormone transcription factor *Bmdimm*-fibroin H chain pathway is involved in the synthesis of silk protein in silkworm, *Bombyx mori*. J. Biol. Chem..

[36] Hu W., Liu C., Cheng T., Li W., Wang N., Xia Q. (2016). Histomorphometric and transcriptomic features characterize silk glands’ development during the molt to intermolt transition process in silkworm. Insect Biochem. Mol. Biol..

[37] Kimoto M., Tsubota T., Uchino K., Sezutsu H., Takiya S. (2014). Hox transcription factor *Antp* regulates sericin-1 gene expression in the terminal differentiated silk gland of *Bombyx mori*. Dev. Biol..

[38] Hui C.C., Matsuno K., Ueno K., Suzuki Y. (1992). Molecular characterization and silk gland expression of *Bombyx* engrailed and invected genes. Proc. Natl. Acad. Sci. USA.

[39] Zurovec M., Yang C., Kodrik D., Sehnal F. (1998). Identification of a novel type of silk protein and regulation of its expression. J. Biol. Chem..

[40] Singh C.P., Vaishna R.L., Kakkar A., Arunkumar K.P., Nagaraju J. (2014). Characterization of antiviral and antibacterial activity of *Bombyx mori* seroin proteins. Cell Microbiol..

[41] Li Y., Zhao P., Liu S., Dong Z., Chen J., Xiang Z., Xia Q. (2012). A novel protease inhibitor in *Bombyx mori* is involved in defense against *Beauveria bassiana*. Insect Biochem. Mol. Biol..

[42] Li Y., Zhao P., Liu H., Guo X., He H., Zhu R., Xiang Z., Xia Q. (2015). TIL-type protease inhibitors may be used as targeted resistance factors to enhance silkworm defenses against invasive fungi. Insect Biochem. Mol. Biol..

[43] Guo P.C., Dong Z., Xiao L., Li T., Zhang Y., He H., Xia Q., Zhao P. (2015). Silk gland-specific proteinase inhibitor serpin16 from the *Bombyx mori* shows cysteine proteinase inhibitory activity. Biochem. Biophys. Res. Commun..

[44] Sakudoh T., Sezutsu H., Nakashima T., Kobayashi I., Fujimoto H., Uchino K., Banno Y., Iwano H., Maekawa H., Tamura T. (2007). Carotenoid silk coloration is controlled by a carotenoid-binding protein, a product of the yellow blood gene. Proc. Natl. Acad. Sci. USA.

[45] Sakudoh T., Iizuka T., Narukawa J., Sezutsu H., Kobayashi I., Kuwazaki S., Banno Y., Kitamura A., Sugiyama H., Takada N. (2010). A CD36-related transmembrane protein is coordinated with an intracellular lipid-binding protein in selective carotenoid transport for cocoon coloration. J. Biol. Chem..

[46] Hossain M.S., Liu Y., Zhou S., Li K., Tian L., Li S. (2013). 20-hydroxyecdysone-induced transcriptional activity of FoxO upregulates brummer and acid lipase-1 and promotes lipolysis in *Bombyx* fat body. Insect Biochem. Mol. Biol..

[47] Yamamoto K., Banno Y., Fujii H., Miake F., Kashige N., Aso Y. (2005). Catalase from the silkworm, *Bombyx mori*: Gene sequence, distribution, and overexpression. Insect Biochem. Mol. Biol..

[48] Ai J., Zhu Y., Duan J., Yu Q., Zhang G., Wan F., Xiang Z.H. (2011). Genome-wide analysis of cytochrome p450 monooxygenase genes in the silkworm, *Bombyx mori*. Gene.

[49] Niwa R., Matsuda T., Yoshiyama T., Namiki T., Mita K., Fujimoto Y., Kataoka H. (2004). CYP306A1, a cytochrome p450 enzyme, is essential for ecdysteroid biosynthesis in the prothoracic glands of *Bombyx* and *Drosophila*. J. Biol. Chem..

[50] Foo C.W.P., Bini E., Hensman J., Knight D.P., Lewis R.V., Kaplan D.L. (2006). Role of pH and charge on silk protein assembly in insects and spiders. Appl. Phys. A.

[51] Andersson M., Chen G., Otikovs M., Landreh M., Nordling K., Kronqvist N., Westermark P., Jornvall H., Knight S., Ridderstrale Y. (2014). Carbonic anhydrase generates CO_2_ and H^+^ that drive spider silk formation via opposite effects on the terminal domains. Plos Biol..

[52] Domigan L.J., Andersson M., Alberti K.A., Chesler M., Xu Q., Johansson J., Rising A., Kaplan D.L. (2015). Carbonic anhydrase generates a pH gradient in *Bombyx mori* silk glands. Insect Biochem. Mol. Biol..

[53] Maxson M.E., Sergio G. (2014). The vacuolar-type H^+^-ATPase at a glance - more than a proton pump. J. Cell Sci..

[54] Wang X., Li Y., Peng L., Chen H., Xia Q., Zhao P. (2016). Comparative transcriptome analysis of *Bombyx mori* spinnerets and filippi’s glands suggests their role in silk fiber formation. Insect Biochem. Mol. Biol..

[55] Lu P., Xia H., Gao L., Pan Y., Wang Y., Cheng X., Lu H., Lin F., Chen L., Yao Q. (2013). V-ATPase is involved in silkworm defense response against *Bombyx mori* nucleopolyhedrovirus. PLoS ONE.

[56] Michalak M., Corbett E.F., Mesaeli N., Nakamura K., Opas M. (1999). Calreticulin: One protein, one gene, many functions. Biochem. J..

[57] Shibata Y., Voeltz G.K., Rapoport T.A. (2006). Rough sheets and smooth tubules. Cell.

[58] Wakefield S., Tear G. (2006). The *Drosophila* reticulon, Rtnl-1, has multiple differentially expressed isoforms that are associated with a sub-compartment of the endoplasmic reticulum. Cell. Mol. Life Sci. Cmls.

[59] Macours N., Hens K. (2004). Zinc-metalloproteases in insects: ACE and ECE. Insect Biochem. Mol. Biol..

[60] Heeneman S., Sluimer J.C., Daemen M.J. (2007). Angiotensin-converting enzyme and vascular remodeling. Circ. Res..

[61] Yu G.C., Wang L.G., Han Y.Y., He Q.Y. (2012). Clusterprofiler: An R package for comparing biological themes among gene clusters. Omics.

[62] Kolde R., Kolde M.R. (2015). Package ‘pheatmap’. R Package.

[63] Mengjun L.G. (2017). Tcseq: Time course sequencing data analysis. https://rdrr.io/bioc/TCseq/f/inst/doc/TCseq.pdf.

